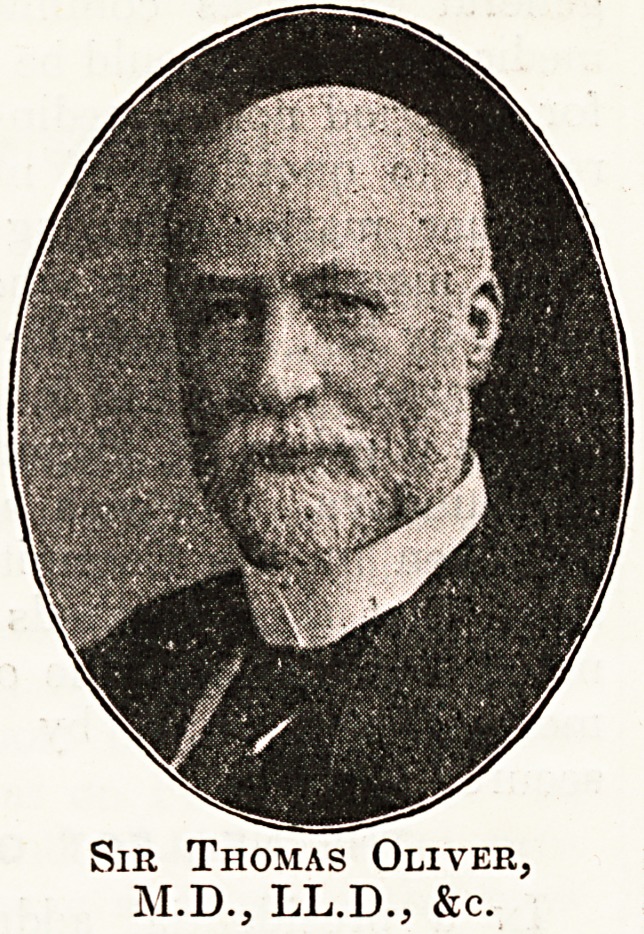# Hospital and Institutional News

**Published:** 1913-07-05

**Authors:** 


### HOSPITAL AND INSTITUTIONAL NEWS.

### THE READERS OF THE PAPERS.

Keith D. Young, F.R.I.B.A.

Sir Thomas Oliver, M.D., LL.D., &c.

### THE OXFORD CONFERENCE: FIRST DAY.

We publish this week a full account of the first day's proceedings of the Conference of the British Hospitals Association at Oxford, which was held on Thursday and Friday last week. The proceedings opened by the Presidential address of Sir William Osler, which preceded a remarkably interesting discussion upon the effect of the Insurance Act. It may be well to point out that Professor Osler's opinions were misrepresented in the general press by the publication of certain rather epigrammatic utterances of his without the context and subsequent explanations. His point clearly was not that the voluntary system should be scrapped as a principle, but that it was hopelessly inadequate unless it included pay wards for patients of moderate means. In the discussion which followed upon the Insurance Act it was decided not to pass resolutions at present, but that the Council of the Association would watch events and submit considered proposals later. It was a matter for great regret that Sir Thomas Oliver was unable to read in person his paper on "Tuberculosis and the General Hospital," as an author's replies to questions arising out of his address are generally among the most interesting things which he has to say. Mr. G. B. Cronshaw read the paper in his absence, and his kindness in stepping into the breach and volunteering for this delicate and rather thankless task is only the most obvious of the many kindnesses which he bestowed upon the visitors. That the Conference was an immense success and, indeed, one may almost say the happiest of the four yet held, was due mainly to his tact, enthusiasm, and geniality. Only those who have organised conferences themselves know how much the outward smoothness of success depends upon unseen and persistent work and worry. The hospitality of Oxford could have had no better exponent, and will be remembered gratefully by all who had the privilege of enjoying it.

### THE SOCIAL SIDE OF THE CONFERENCE.

Nobody who had the privilege, as was the case with some twenty or more members of the British Hospitals Association, of staying at Queen's College during the Oxford Conference will have the least hesitation in saying that its social side was as valuable as its papers and discussions. Mr. Thies, who, as usual, took endless pains over its details, and indeed paid three visits to Oxford before the Conference to discuss its arrangements, was told by many members how grateful they were for their opportunities of being introduced to other hospital men, whom, but for the Conference, they would never have known personally. Among the unofficial subjects debated between the sessions, or during the delightful evenings in the smoking-room at Queen's, was, of course, the Uniform System of Accounts. It is a subject which generally proves suggestive. The course of criticisms, comparisons, and individual methods, which came in volleys from excited speakers, was as interesting as it was amusing. Its moral was the practical one, that it is hopeless to expect an absolute uniformity, and that the recommendations for changes in the system must be general as extending a principle, rather than particular as to the invariable application of it. Individual institutions must fill out the skeleton in their own way; though the secretary who happens to be a keen accountant will find how much more can be done than the secretary who rather regards all accounts as a bugbear. From the personal explanations given of why this entry was made, or that omitted, provincial and London men alike saw that there was more to be said for each other's methods than they had supposed hitherto. This feeling, which is the beginning of all statesmanship, can be produced only by social intercourse. That is an important reason why these conferences have so much value.

### THE SPEAKERS AND THE DEBATES.

The Conference at Oxford of the British Hospitals Association this year differed in one important respect from its predecessors. The speeches were better. If we look at the subjects which formed the texts of debate, we shall see that two were general, and one particular, and technical in the narrow sense of the word. Though the speeches this year were all good, on the whole, the debate which seemed most to interest every member of the audience was that which followed Mr. Keith Young's concise and suggestive paper on "The Upkeep of Hospitals." It was a paper on the importance of "Wrinkles," and the debate was a lively exchange of ideas upon these. Floor materials, the effect of avoidable turnings in pipes upon the coal bill, the daily reading of meters, the destruction of brass lacquer by "polishers," in short, details of all kinds, on which experience alone can decide, were taken up with the greatest eagerness. "What is your experience?" and the no less eager "my experience undoubtedly is" made the debate a lively and arresting one. Every intelligent participator must have felt that it was a pity that it could not be a weekly, instead of an annual, discussion. As a matter of fact it can. The "Bureau of Information" in "The Institutional Worker" Supplement of The Hospital exists to provide a weekly exchange of experience on matters of technical interest and detail. The rules for correspondents could not well be simpler than they are, and we hope that the eighty or more hospital men who were present at this debate will remember that "Our Bureau of Information" provides the means of continuing it throughout the year. The Bureau has one advantage over the debate. Printing places the worst speaker on a footing with the best, and in reading attention is not distracted by bad articulation or the shuffling of feet. Finally, let every person with a question to which he wants an answer remember, that for one intelligent enough to ask it there are twenty who want to know the answer.

### THE EFFECT OF PAY WARDS ON SUBSCRIPTIONS

The emphasis placed by Sir William Osler on the importance of having pay wards attached to the voluntary hospitals has awakened a responsive echo in Cardiff, where Mr. Isaac Samuel, J.P., chairman of the finance committee of King Edward VII.'s Hospital, has been interviewed by the *South Wales Daily News*. "I have advocated," he is reported, "paying wards for a long time for those who cannot honestly go to the hospital and cannot pay the high fees of specialists. It is done at Liverpool, at Weston, and in some London hospitals. Why not in Cardiff?" Our readers will remember that Mr. Leonard D. Rea, the secretary-superintendent, described in our columns the system of patients' payments on its introduction at King Edward VII.'s Hospital. That, of course, was a different thing, as its aim was rather to benefit the hospital than to meet the needs of a new class of patient. We mention Mr. Samuel's opinions, however, as an encouraging sign of the latent readiness to build pay wards which has long existed among certain, if not all, hospital managers. A favourite objection, which all American experience goes to disarm, is that pay wards tend to reduce voluntary subscriptions. As a matter of fact they are the recruiting ground of new subscribers and large donations, and are the best—that is, the most lucrative—advertisement that a hospital can have. A rich patient who has come successfully through an illness or operation in hospital is found never to forget it, and very commonly, on noticing something wanting during his stay, sends a cheque to supply the need on leaving. Even hospital secretaries hardly realise how many rich and well-to-do people there are who have no idea what the inside of a hospital looks like. Pay wards, by extending the benefits of the institution, extend its reputation. The objection that they reduce subscriptions is found to be the direct opposite of the truth. The fact, urged by another speaker, that the younger medical men dislike pay wards has been adjusted in countries where they have been introduced.

### PANEL SERVITUDE ON SUNDAY.

The London Insurance Committee at its meeting last week adopted the recommendation of its general purposes committee that four part-time medical officers should be engaged as an experiment for a period not exceeding six months to assist in regard to questions of malingering or invalidism. Another matter affecting the profession, at least those members of it who are on the panel, was raised by Mr. P. Rockliffe, who moved for a return, and stated that he had found that out of 128 doctors on the panel in St. Pancras 89 gave no attendance on Sundays. He was anxious that steps should be taken by the Committee to let doctors understand that their methods of procedure were being noted, for that was the only way in which proper medical attendance by panel doctors could be secured.

### THE NEGLECT OF BRITISH SPAS.

In a presidential address delivered before the Yorkshire Branch of the British Medical Association, Dr. Charles Gibson has recently deplored the fashion of going abroad to Continental spas, when every possible advantage thus derived can equally well be secured at home. The complaint of neglect and under-valuation of our British spas is a very old one and one full of substance; for practically every disease which can be benefited by foreign spas can be just as adequately treated in Britain. This tendency to go abroad is not easy to explain satisfactorily: it is more expensive, less convenient, and no more efficacious, yet still it continues to exist. One factor which conduces to it may be the pretentious pomposity of the medical treatment. At many foreign spas the visitor is supplied with wonderful charts purporting to represent the thousandth milligram of the salts in the water he drinks; the exact number of calories in the food he eats; the analysis of two or three score ingredients in the excretions of his kidneys, none of which are of the very slightest moment or importance; and, in fact, there is a great display of pseudo-scientific elaborateness which impresses the visitor with the idea that foreign doctors know "so much more" than British ones. We do not advocate the imitation of these tactics by our home physicians, but we do believe they explain to some extent the seductiveness of foreign spas. Meanwhile, we can agree with Dr. Gibson concerning the merits of the Harrogate waters, which become steadily more popular every year.

### SANATORIUM BENEFIT SUSPENDED IN DUBLIN.

As we pointed out three weeks ago, panel servitude in Ireland has virtually broken down, owing to the refusal of the medical men to accept the proposed terms for certification. By ten votes to one a recent meeting of the Dublin County Borough Insurance Committee passed a resolution, the result of which is the suspension of sanatorium benefit in the county and city of Dublin. The resolution stated that the Committee was of opinion that sanatorium benefit could not be carried out satisfactorily if applicants for benefit were to be compelled to secure certificates from some one of the thirteen "medical advisers" appointed by the Insurance Commissioners to dispense certificates for the county and city area. The Irish doctors' victory in the question of certification appears to be complete, and the Act to be paralysed in consequence.

### THE INSURANCE COMMISSIONERS' ACTION.

The immediate result of the Committee's resolution was to produce a letter from the Insurance Commissioners in Dublin. In the course of this they state: "As there is no obligation upon an applicant for sanatorium benefit to obtain a certificate from any particular doctor, the procedure generally adopted . . . is that the applicant is examined by his own medical attendant, . . . who furnishes a preliminary report on Form Med. 2, a subsequent report being made by the Committee's medical adviser on Form Med. 4. In accordance with the demand of the medical profession, the Commission have sanctioned the payment by Insurance Committees of a fee of 5s. for each examination and report on Form Med. 2. . . . One of the conditions on which medical advisers were recently appointed by the Commission was that they should, free of charge, examine and make the preliminary reports in the case of any . . . persons who might apply for sanatorium benefit, if such reports were required. It was thus intended to relieve the Committee of the necessity of paying in fees moneys which could be more advantageously expended on the actual treatment." The letter concludes with the following vaguely worded threat: "Having regard to the terms of the resolution referred to, I am to request that the Commission may be furnished at once with a detailed statement of the sums to the credit of the Committee's account, and of the liabilities as at the 19th inst., and I am to point out that, should the policy outlined in the resolution be adopted by the Committee, the Commission must take effective steps" to secure sanatorium benefit.

### WHAT DOES SANATORIUM BENEFIT MEAN?

As a good many insured persons have been finding out since last July, "sanatorium benefit" does not necessarily mean treatment in a sanatorium at all; the term, which is misleading and undesirable, includes also dispensary treatment and panel doctoring in the patient's own home. A Parliamentary Paper (Cd. 6884) just issued contains some interesting figures on the administration of sanatorium benefit for the first nine months of its operation. (Sanatorium benefit, it will be remembered, began on July 15, 1912; not on January 15, 1913, as was the case with the other medical benefits.) In England 14,624 patients received sanatorium benefit during the period under review; just over half of them went to residential institutions, and just under half had the other sorts of benefit. In Scotland 2,014 qualified; just over three-quarters got into residential institutions, and only 5 per cent. of them attended dispensaries; the other 20 per cent. received domiciliary treatment. In Ireland two-thirds of those found suitable for sanatorium benefit went to sanatoria, and tuberculosis dispensaries seem to be at as great a discount as in Scotland. In Wales, on the other hand, this is the favourite form of benefit: one-third of the 1,162 patients went to sanatoria, two-fifths to dispensaries, and the rest had domiciliary care. For all this the Welsh methods seem much more expensive, as the average cost per patient was practically twice what it was in Ireland, and considerably greater than in England and Scotland. Why sanatoria should work out cheaper than dispensaries and domiciliary treatment passes our comprehension; the Parliamentary Paper suggests no reason for it, and the Treasury might well investigate the matter, which is certainly one that requires explanation.

### ASYLUMS BOARD ESTIMATES: EFFECT OF REVISED SALARIES.

The finance committee of the Metropolitan Asylums Board has submitted its estimates for the half-year ending March 31, 1914. The net total required, excluding the expenditure on the Downs Sanatorium which will be refunded to the Managers, is £572,330, or £7,315 more than for the corresponding period in 1913. The increase in the ordinary expenditure of £16,675 is chiefly due to the revised salaries and wages scale, which our readers will remember, and also to a rise in the coal bill under the new contracts. By drawing on the balances, however, and thanks to increased rateable value, the committee does not recommend an increase in the rate in the pound for common charges.

### INCREASED SALARIES AT CAMBERWELL INFIRMARY.

Camberwell Board of Guardians have decided to increase the salary of Dr. W. J. C. Keats, medical superintendent at the infirmary, from £475 to £575, with emoluments valued at £170. The additional £100 a year is a tribute on the part of the Guardians to the work which Dr. Keats has accomplished at the infirmary. Ten years have elapsed since he last received any extra remuneration for his arduous duties, which, in the intervening years, have been still further increased. That the Guardians recognise his skill and ability is evinced by the fact that only two members voted against the extra salary. The Board has been unable to obtain applications for the vacant post of junior assistant medical officer at the infirmary. Dr. Keats pointed out that twenty-two years ago they had thirty-nine good applicants for the post at £130 per annum, but at the present time they could not get one at a salary of £150. The Board decided on an all-round increase of salaries. The assistant medical superintendent is in future to receive £230, rising to £260; the doctor at the Children's Home, £200, rising to £230; at Gordon Road Workhouse, £180, rising to £190; fourth assistant medical officer, £160, rising to £170; and the junior medical officer, £140. The latter post is to be open to candidates of either sex.

### A NATIVE KING VISITS LEICESTER INFIRMARY.

The Leicester Royal Infirmary on Saturday last received a visit from the Kababa or King of Uganda and his chiefs, who are now on a visit to England. As the guests in Leicester of Mr. Theodore Walker, a member of the board of governors of the infirmary, it was fitting that they should be made acquainted with one of the most progressive of the English voluntary hospitals, and the King, who, we learn, is deeply interested in institutional work, expressed his appreciation of what he saw by numerous inquiries. He also signed the visitors' book.

### ATTENDANTS' PETITION AT CARDIFF.

The visiting committee of Cardiff Mental Hospital has agreed to "recognise" the National Asylum Workers' Union. This was decided on at a meeting, over which Dr. James Robinson presided, when a petition from the male attendants was presented asking for recognition, and declaring that their chief concern was to safeguard the position of the local secretary. The female attendants were not on the list. Dr. Goodall, the medical superintendent, admitted that he did not know what "recognition" meant. He did not think that any secretary of the Union would be molested, provided he complied with the regulations of the institution. Councillor Jones thought that they had agreed to the right of servants to form a Union, and he was in favour of its recognition.

### PRESIDENT POINCARE AND THE FRENCH HOSPITAL.

On Wednesday last week President Poincaré visited the French Hospital and Dispensary in Shaftesbury Avenue. M. Ernest Lazarus-Barlow, the hospital's president, received M. Poincaré, and, in leading him to the committee room, where the medical and nursing staffs were represented, made a brief speech in which he alluded to the gratifying contribution of £8,000 from the French Government, which had enabled them to found the French Convalescent Home at Brighton. In his reply the President drew attention to the visits which M. Loubet and M. Fallières had paid to the institution. M. Poincaré then decorated Mr. W. H. Clayton-Greene, who has been for six years a member of the honorary surgical staff, with the Cross of the Legion of Honour.

### SANATORIUM DOCTOR RECEIVES AN APOLOGY.

The West Suffolk County Council has tendered its sincere apologies to Miss Jane Walker, the well-known lady doctor, and agreed to pay £65 for costs as the result of a writ and an interim injunction which she had issued against the Council. At Nayland, in Suffolk, Miss Walker has tenanted a farm where she carried on for tuberculosis patients the "Maltings Farm Sanatorium." According to the evidence, on May 28 last, without communicating with her, the Council published a notice to the effect that it was proposed to hold an inquiry with respect to the Sanatorium. Miss Walker, considering that this notice was likely to injure the Sanatorium's reputation and her own, and that the inquiry ought not to be held, issued a writ against the Council, and claimed a declaration that the Council had no power to hold an inquiry, an injunction to restrain them from doing so, and an injunction to restrain them from printing or publishing any notice that such an inquiry would be held. An interim injunction in terms of the writ was moved for on June 13, and the motion by arrangement stood over till last week, when, as the result of correspondence between the parties, the above friendly settlement was carried out. Apart from the sympathy which an individual always receives from the public when in litigation with a public body, institutional managers will be glad to note that this action is likely to restrain public bodies from making rash announcements concerning institutions without communicating with the responsible officer. How any body could proceed in this way at all is sufficiently astonishing.

### SOME RECORD IMPROVEMENTS IN 1912.

The annual summary of the Registrar-General's vital statistics for the past year in England and Wales discloses some really remarkable figures. There was, of course, the usual fall in the birth-rate, though not a large one; and there was an actual increase in the marriage-rate, also a small difference compared with the previous year. The death-rate was as much as 1.3 per 1,000 below that of 1911. Furthermore, this satisfactory death-rate was conduced to very largely, probably mainly, by a diminution in infantile mortality; in other words, it was the nation's children which were saved rather than the great-grandparents. Infantile mortality in the first year of life was ninety-five per thousand, as against one hundred and thirty for 1911, and an average of one hundred and twenty-five for the past ten years. Doubtless the cold wet summer prevented a great deal of infantile diarrhœa last year; and the inflation of the 1911 figures above the decennial average was probably due to the hot dry summer of that year. It is hardly necessary to say that ninety-five per thousand is easily the lowest figure of infant mortality on record. The large towns have done especially well in reducing their mortality bills. Urban life is still less healthy than rural; but the difference is lessening. In London there was a much higher marriage-rate than for the country at large; in fact, a higher rate than has been recorded since 1899. If 1913 improves on the records of 1912, practical hygienists may well feel full of pride and encouragement.

### THE DEFINITION OF IDIOCY.

The Mental Deficiency Bill has raised a new interest in the accurate definition of such terms as "mentally deficient" and "idiot." How, the opponents in particular of the Bill have been asking, is it to be decided who comes under such apparently loosely classified terms? At a recent meeting of the Norwich District Ladies' Association of the Royal Eastern Counties Institution for Idiots, Imbeciles, and the Feeble-Minded, Mr. Arthur M.-

Samuel, the Lord Mayor of Norwich, did good service in reminding his audience of what the word idiot meant to Athenians in the days of Pericles. After remarking that it was difficult to know who was quite normal, Mr. Samuel remarked that the old Athenian word meant one who did not take part in municipal government. If that definition were adopted, the speaker humorously added, all people who did not put up for the Town Council were idiots. The late Samuel Butler, humorist and philosophical writer, in supporting his favourite dogma of Laodiceanism, once remarked that all those who blew either too hot or too cold, or, in other words, differed too greatly from their fellows, were, in the strict etymological meaning of the word, idiotic. A later writer, taking up this point, has stigmatised the specialist in the same way. Definition, then, is a matter of difficulty, and probably the Mental Deficiency Bill is wisely framed in preferring the certificates of two medical men to any verbal attempt at it. The institution on whose behalf the above speech was made is preparing for extension, which will consist of a new block providing for seventy additional patients.

### DEATH OF MR. ALFRED WILLETT, F.R.C.S.

Mr. Alfred Willett, F.R.C.S., formerly a member of the Council of the Hospital Sunday Fund, and one of the visitors for King Edward's Hospital Fund, has died at the age of seventy-six. He was almost equally well known as a surgeon and as a man interested in hospital administration. Among his several appointments at St. Bartholomew's Hospital we may mention that he was Lecturer on Surgery and Warden of the College. He was, or had been, surgeon also to St. Luke's, Queen Charlotte's, and the Foundling Hospitals. His administrative interests, apart from his connection with the two great Funds above mentioned, were represented by his respective posts as director and member of the board of the Royal Sea-Bathing Hospital, Margate, and the Metropolitan Convalescent Institution. He retired from the Council of the Royal College of Surgeons ten years ago, and had been living at his Sussex house where he died.

### ALEXANDRA DAY, 1913.

There can be little doubt that Alexandra Day is establishing itself in the affections of the public, and there were few individuals to be seen on Wednesday last week who had not shown their interest in the cause of charity, or purchased immunity from further solicitation, by wearing one of the roses which were being sold. The two ways of making such a form of charitable effort successful seem to be to offer for sale a decorative badge which can be manufactured very cheaply, and to make the sellers attractive. It is a sound principle to give the donor something for his money, and, as there was never probably a time when badges, decorations, and symbolic ornaments were more popular than at present, a purchase which could be displayed was bound to succeed. The day was kept in many parts of the country, and the net total realised by its means must have been very great.

### NEW CHIEF MEDICAL OFFICER TO THE POST OFFICE.

Dr. John Sinclair has been appointed by the Postmaster-General to be Chief Medical Officer to the Post Office in place of Dr. A. H. Wilson, who is retiring under the age limit. Dr. Wilson, who was a student at St. George's Hospital, and afterwards house sugeon there, took the Conjoint Diploma in 1876. His successor, Dr. Sinclair, who has been Second Medical Officer to the General Post Office, qualified in 1882 and graduated in medicine at Durham in 1900. A London Hospital man, he was formerly house physician there, and is the joint author of a paper on "Telegraphists' Cramp," which appeared in the *Lancet* last year.

### DEATH OF A HOSPITAL SECRETARY.

We have to record the death of Mr. William Thraves, the secretary of the Chesterfield and North Derbyshire Hospital, at the age of fifty-seven. In the beginning of last autumn he went to Bournemouth hoping to recuperate, for he had been in weak health for some months before, and though he appeared to benefit by the change his resumption of work was followed by a collapse. The cause of death was cancer. Appointed secretary to the Chesterfield Hospital in 1902, this was, we believe, the first responsible hospital appointment that he had held, though his interest in hospital work had always been keen. Having spent his early years in Chesterfield he began life as a waggon ticketer in a coal and iron company, which he entered in 1871. His abilities gained him promotion till he was placed in charge of the ledger department, which he left only to become a hospital secretary. During his period of office the medical wards, given by the late Mr. Eastwood, were added, and a nursing home has been built, by which additional beds have been available for patients. To his weekly routine work he added frequent Sunday speeches on behalf of the institution. A teetotaller, non-smoker, and strong "Liberal," it used to be said of him that he had held every office open to a Methodist layman. The hospital now consists of 120 beds, and 1908 is the official date of its latest extension. Mr. Thraves had an assistant secretary, Mr. George Lewcock, on whose shoulders much responsibility and work have fallen during Mr. Thraves's protracted illness.

### THE RANGE OF PANEL SERVITUDE.

An insured person, in the area of the Surrey Insurance Committee, has complained that, when, as the result of having three teeth extracted by a dentist, hæmorrhage became serious and the panel doctor was asked to attend, he refused, to do so on the ground that dental benefit did not come under the Act. The doctor said that if the dentist could not stop the bleeding he must call in a doctor at his own expense. The medical benefit sub-committee, concluding that it is not their business to decide the range of medical service, recommend that the Insurance Commissioners be asked to give a definite ruling on the point raised by this case. For the consolation of the insured person apparently, they state that in their view the practitioner under the circumstances should have attended the patient, and then referred any point in dispute to the local medical committee under Article 53 of the Regulations.

### GIFT OF RADIUM TO THE MIDDLESEX HOSPITAL.

The Middlesex Hospital has received £1,000 from Sir Alfred Pearce Gould for the purchase of radium to be used for the treatment of patients who suffer from malignant disease. Mr. Clare Melhado, the secretary superintendent, hopes that the example shown to the public from so eminent a medical source will lead others to subscribe towards an adequate fund for the purchase of radium. In spite of the gifts recently made the supply available is very small, and the demand is far in excess of it.

### CHARGE OF IMPERSONATING A PHARMACIST.

A chemist charged with forging an application to the Pharmaceutical Society of Great Britain for restoration to the Register of Chemists and Druggists, and further with wilfully procuring himself to be registered under the Pharmacy Act of 1868 by making and producing a false and fraudulent representation in writing that he was one Frederic Lancelot Roberts, was committed for trial at Bow Street Police Court last week. It was alleged on behalf of the Pharmaceutical Society that the defendant had been personating his brother, Frederic Roberts, whose name had been struck off the Register in 1899, after two letters, which had been sent to his last known address in Forest Gate, had been returned unopened. In 1908 the defendant was found dispensing drugs at a Woolwich drug stores, and in answer to the inquiries of the Pharmaceutical Society's inspector gave himself out to be Frederic Lancelot Roberts, and had that name restored to the Register. Since then, it was stated, the defendant had been signing documents in his right name of Edward William Roberts. The defence was reserved, but bail of £100 was allowed in the defendant's own recognisances.

### ST. GEORGE'S AND WESTMINSTER HOSPITALS PROPOSED AMALGAMATION.

A joint sub-committee of the two institutions has been sitting recently to investigate the basis of the proposed amalgamation and the question of removal to another site. The report of this committee is expected shortly, when the matter will no doubt come before the governors of the respective institutions in due course.

### THE COST OF THE MAUDSLEY HOSPITAL.

The London County Council are finding that the original estimate of the cost of the new Maudsley Hospital is likely to be considerably exceeded. In December the Council authorised expenditure on capital account of £56,000 in respect of the erection and equipment of the new hospital. They have now obtained from selected firms seventeen tenders for the erection of the building, but the total of the lowest tender, which it is proposed to accept, is £43,248, whereas the provision made in the Council's vote in respect of the work tendered for was only £39,817, a figure based upon the asylums engineer's estimate of cost.

### DISPENSARIES AND THE INSURANCE ACT.

As soon as the Insurance Act came into force the voluntary hospitals were compelled to come to its rescue by pointing out that its provisions did not include institutional treatment, thus, whether for insured persons or not, serious cases and practically all surgical cases fell to the hospitals as before. The much-vaunted benefits of the Act did not cover serious illness. Their area was restricted to the minor ailments of an insured person. His dependants were excluded from its operation, with the result that just as the hospitals had to save the Act by accepting all the difficult work, the provident dispensaries have also hardly less work to do, since, though minor ailments have been their special care, the dispensary class consists mainly of women, and children up to sixteen years of age. The charitable public has not realised this, with the result that the Metropolitan Provident Medical Association, of which Mr. Claude G. Montefiore is chairman, is appealing for funds, and it is interesting to see how the Act has affected their institutions. At the close of 1912, it appears, there were about forty provident dispensaries in London, with some 140,000 members—men, women, and children. From one quarter to one third of these have become insured under the Act, leaving 100,000 on the books in London. This number is expected to reach, and even to surpass, its former figure, and help is required to tide the dispensaries over the difficult interval. That the dispensaries and the voluntary hospitals are still both needed shows how narrow is the area of medical benefit covered by the Act. The women and children, broadly speaking, are not touched by it at all.

### THIS WEEK'S DRUG MARKET.

There has been some indication of a renewal of interest in quinine, but in the absence of confirmation of the report that a definite settlement of the negotiations between growers of cinchona bark and makers of quinine had been arrived at buyers are disposed to be cautious. It is difficult to know what to advise, but an advance in the price of quinine would appear to be more likely than a material decline. The advancing tendency in the price of cod-liver oil is maintained, but very little business has been done. Citric acid has been in good demand and the price tendency is in an upward direction. Oil of almonds and oil of cloves are slightly lower in value. At the recent public sale of drugs at Mincing Lane, Cape aloes, cardamoms, ipecacuanha, and sarsaparilla sold at rather lower prices; Jamaica honey was rather dearer.

## Figures and Tables

**Figure f1:**
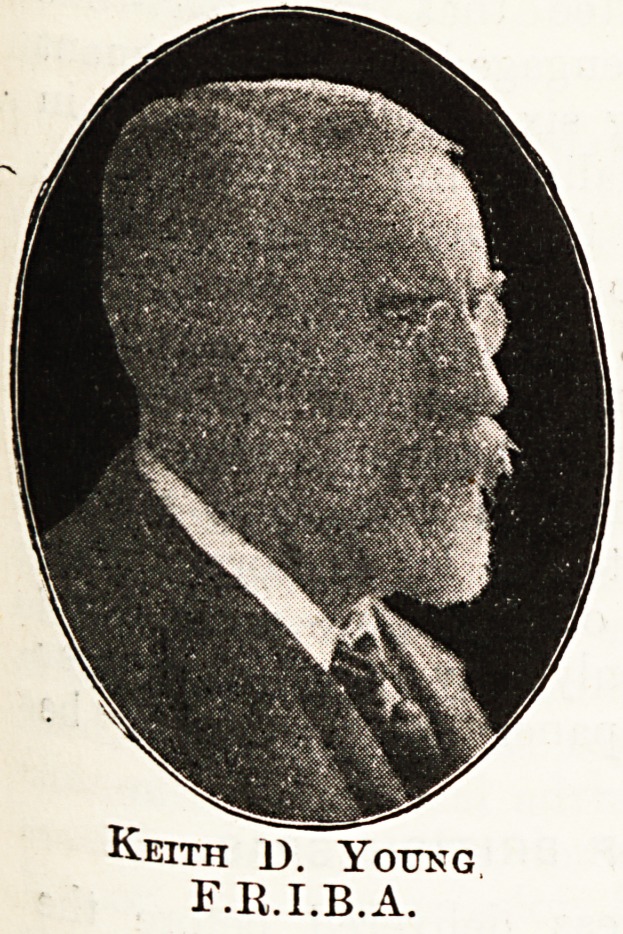


**Figure f2:**